# Mutual obligation, shared responsibility agreements & indigenous health strategy

**DOI:** 10.1186/1743-8462-3-10

**Published:** 2006-09-25

**Authors:** Ian PS Anderson

**Affiliations:** 1 Onemda VicHealth Koori Health Unit, Centre for Health and Society, School of Population Health, University of Melbourne, Parkville, Melbourne, Victoria, Australia

## Abstract

Since 2004 the Howard Coalition government has implemented a new policy framework and administrative arrangements as part of its program of reform in Indigenous affairs. In this paper I will describe both the parameters of this reform program and review the processes established to support the implementation of national Indigenous health strategy. In particular, I will consider both the shift from a policy framework based on 'self-determination' to one based on 'mutual obligation', and the implementation of Shared Responsibility Agreements (SRAs) that are based on the latter principle. I will use the example of the Mulan SRA to illustrate the difficulties in articulating the 'new arrangements' with current approaches to Indigenous health planning and strategy implementation. I conclude that 'new arrangements' pose a number of problems for Indigenous health planning and strategy that need to be addressed.

## Background

In 2004 the Howard Coalition government embarked on a radical reform program in Aboriginal affairs, beginning with the announcement on 15 April 2004 of its intention to abolish the Aboriginal and Torres Strait Islander Commission (ATSIC). [1] The Commission had been established under legislation passed in 1989, which merged the program responsibilities of the Department of Aboriginal Affairs and the Aboriginal Development Corporation. ATSIC's original structure included regional councils that had a role in regional resource allocation. A board of commissioners, which were elected from the pool of regional councillors, had oversight of ATSIC's national programs and were also responsible for the provision of policy advice to the Minister for Aboriginal and Torres Strait Islander Affairs. [2,3]

The Australian government's decision to abolish ATSIC was announced in the context of the run-up to the 2005 federal election. Only a few weeks earlier, the opposition leader of the Australian Labor Party, Mark Latham, launched a similar policy – in which ATSIC was to be abolished but replaced with a new regionalised body. [4] Prime Minister Howard's announcement, in this context, did give the appearance of policy on the run. However, this government had been in longstanding conflict with ATSIC on a number of fronts. For instance, ATSIC resisted the Howard government's move away from a rights-based agenda in Aboriginal and Torres Strait Islander affairs to one based on 'practical reconciliation'. The Commission had also faced persistent allegations of corruption and its critics charged it with the failure to improve outcomes for Indigenous Australians. [2,5,6]

In 2002 the Australian government commissioned a review of ATSIC. The review panel recommended the retention of the Commission, but with significant structural reform. [7] The government, however, did not wait for the review to be completed before tackling structural reform – from July 2003 the commission was split into two arms, an elected and an administrative arm (Aboriginal and Torres Strait Islander Services, ATSIS), in order to create a 'separation of powers'. [2] Despite these measures the Australian government, by April 2004, had come to the:

*very firm conclusion that ATSIC should be abolished and that it should not be replaced, and that programmes should be mainstreamed and that we should renew our commitment to the challenges of improving outcomes for indigenous people in so many of those key areas*. [1]

In the months that followed, ATSIC's programs were reallocated to Australian Government departments and portfolios (see Table 1); the National Indigenous Advisory Council was established and the development of 'Shared Responsibility Agreements' (SRAs) was initiated. [8-10]

**Table 1:** The transfer of ATSIC programs to Australian government departments and portfolios. Source: [8]

**Australian Government Portfolio/Department**
ATSIC program transferred
**Department of Immigration and Multicultural and Indigenous Affairs**
Indigenous rights
International Issues
Public Information
Repatriation
Planning and Partnership Development
Community Participation Agreements
Native Title and Land Rights
Indigenous Women's Development
Indigenous Women
Office of Torres Strait Islander Affairs

**Immigration and Multicultural and Indigenous Affairs Portfolio**
Indigenous Land Fund
Indigenous Land Corporation
Torres Strait Regional Authority
Registrar of Aboriginal Corporations
Regional Councils

**Aboriginal and Torres Strait Islander Services**
ATSIC Housing Fund
ATSIC's functions under the Native Title Act
Business loans and program grants made by ATSIC before 1 July 2003
Administration of the Regional Land Fund

**Department of Employment and Workplace Relations**
Community Development and Employment
Business Development program

**Employment and Workplace Relations Portfolio**
Indigenous Business Australia

**Department of Family and Community Services**
Community Housing and Infrastructure
Family Violence
- Family Violence Prevention (shared with Attorney-General's Department)
- Family Violence Partnership program

**Family and Community Services Portfolio**
Aboriginal Hostels Limited

**Department of Communications, Information Technology and the Arts**
Arts, Culture and Language
Broadcasting
Sport and Recreation

**Department of Health and Ageing**
Effective Family Tracing and Reunion Services

**Department of the Environment and Heritage**
Maintenance and Protection of Indigenous Heritage

**Attorney-General's Department**
Legal and Preventative
Family Violence Prevention (shared with Department of Family and Community Services)

**Education, Science and Training Portfolio**
Australian Institute of Aboriginal and Torres Strait Islander Studies

**Finance and Administration Portfolio**
Office of Evaluation and Audit

In the last two years over 100 SRAs have been developed (for a comprehensive register of these agreements see [11]). The critique of SRAs has focused on linking a discretionary benefit to basic civic rights; concerns about the capacity to evaluate them; the potential of SRAs to produce health outcomes and their underpinning ethics [12-14]. Notwithstanding the concerns raised about the SRAs, the current Aboriginal affairs minister, Mal Brough, remains positive about their contribution, even whilst announcing a review of their implementation [15].

The idea of 'mutual obligation' underpins this new approach to Indigenous affairs. This policy construct has been used to frame the development of the SRAs – which I illustrate below using the 'Mulan agreement' as a case study. This new policy framework in Indigenous affairs departs from a regime based on the notion of 'self-determination' (first introduced to national policy in 1972). [3,16] It is arguable as to whether this shift in policy has been based on an informed review of trends in outcomes or even an analysis of the application of the principles of self-determination to policy and service delivery. Here, however, it is my intention to describe the contours of these reforms – not to evaluate the basis for them.

When ATSIC was established, Aboriginal health was one of its program responsibilities. However, the responsibility for the administration of this program was transferred to the health portfolio in 1995, following the evaluation of the National Aboriginal Health Strategy (endorsed 1989) and lobbying by the Aboriginal community controlled health sector. [17,18] Since then, considerable focus has been given to the development of mechanisms to coordinate the implementation of Aboriginal health strategy and planning within the health sector. This has resulted in the development of multi-sectoral intergovernmental Framework Agreements, a national approach to performance management and the development of regional planning forums. Strategy development has focused on both the development of the health care infrastructure (with a priority for primary health care) and biomedically defined priorities. [19-21]

Following the transfer of the Aboriginal health program, ATSIC continued to play a significant role in inter-sectoral strategy in Indigenous health. ATSIC's environmental health and housing programs addressed significant determinants of Indigenous health. Consequently, the respective roles, responsibilities and working relationship between the health portfolio and ATSIC were defined in a Memorandum of Understanding that was agreed to in 1995. [17]

In the sections below I describe the parameters of this reform program and the mechanisms currently associated with Aboriginal and Torres Strait Islander health strategy and planning. The implications of the broader reform agenda in Indigenous affairs for strategy and planning in health is considered in the concluding discussion in this article.

## Mutual obligation

Mutual obligation has been a contested concept in Australian public policy over the last decade, particularly in debates on welfare reform. Although the idea has a much longer history, emerging originally from eighteenth and nineteenth century debates about the social contract that binds citizens and state, it has been mobilised in this context by what has been constructed as the of 'problem of welfare dependence'. [22,23] Some commentators claim that welfare dependence is not explicitly defined in the Government policy, lending it to ambiguous or contradictory interpretations. [23] Nevertheless, it can be inferred that welfare dependence is seen to emerge when individuals on income support rely on governments, not so much because they lack the capacity for self-reliance, but the will. [23] Accordingly, Prime Minister Howard outlined in an address to the 'Australia Unlimited Roundtable' in 1999:

*Just as it is an ongoing responsibility of government to support those in genuine need, so also is it the case that – to the extent that is within their capacity to do so – those in receipt of such assistance should give something back to society in return*. [24]

According to the Office of Indigenous Policy Coordination (OIPC), governments and Indigenous people "have rights and obligations and all must share responsibility". [25] The problem, as they see it, being that:

*Despite best intentions, over the last 30 years, programs and services have been delivered in ways that increase dependence on government and welfare, rather than building on the creativity and self-reliance of Indigenous people*. [25]

The assumptions that underlie this position and the application of 'mutual obligation' in Aboriginal affairs have been contested, as they have been in the broader welfare sector. Some Indigenous leaders, such as Noel Pearson, have advocated positions that converge with this idea. He argues, for instance, that the "provision, without reciprocity, of income support to able bodied people of working age can be seen as 'negative' welfare, including passivity and dependence." [26] Nevertheless, he cautions in an article with Patrick Dodson against the potential 'dangers' of an uncritical application of 'mutual obligation' – particularly the extent to which the idea can be potentially used to unnecessarily intervene in private lives and in particular social and economic behaviours. [27]

In the public debate on Aboriginal and Torres Strait Islander self-determination there has been a tendency to focus on ATSIC as the sole institutional manifestation of this policy [18]. It is significant in this regard that the new policy structure, the National Indigenous Council, was established by Ministerial appointment and not elected representation. [9] However, at its broadest, the policy of self-determination has been realised through a number of different processes. This includes the development of participatory policy processes, Indigenous management of services (such as primary health, legal, housing and related community services) and, more generally, the promotion of Indigenous leadership and decision-making. [16,28]

Notwithstanding the broader approach to the application of principles of self-determination, Australian Government strategy continues to rely to a significant extent on the delivery of Indigenous-specific health and community services through Aboriginal cooperatives that have locally elected boards of management – the ACCHs. [19] In 2003, prior to the abolition of ATSIC, all Australian governments signed onto the National Strategic Framework for Aboriginal and Torres Strait Islander Health. This in effect locks in the Howard government, at least in the medium term, to a strategy in which key result area one:

*aims to continue support for adequately resourced, well-planned ACCHSs. It advocates partnerships between community controlled health services and mainstream services to ensure that Aboriginal and Torres Strait Islander communities have access to the full range of services expected within the comprehensive primary health care context. It supports the fundamental principles of community decision-making, influence and control over the way health services for Aboriginal and Torres Strait Islander peoples are managed and delivered*. [21]

## Reforms in indigenous program administration

On 30 June 2004 the Minister for Indigenous Affairs announced, "more than $1 billion of former ATSIC/ATSIS programs have been transferred to mainstream Australian Government agencies and some 1300 staff commence work in their new Departments as of tomorrow". [29] The new administrative arrangements for these programs is summarised in Table 1. However, there is more to this reform program than the disestablishment of the Australian government's Indigenous bureaucracy. A number of the reforms also draw upon current debates in Australian public policy and internationally about the development of effective whole-of-government responses or "joined up government" recognising that "Both the effective development of policy, and the efficient delivery of the services that are the concrete manifestation of policy, are equally hinder by departmentalism". [10,30] To that end the reforms in Indigenous program administration include the creation of central co-ordination mechanisms such as:

• A ministerial taskforce that operates as a cabinet committee, providing collaborative leadership and setting strategic directions.

• A Secretaries group supporting ministerial decision-making, coordinating across government agencies, and overseeing annual reporting.

• An Office of Indigenous Policy Coordination (OIPC), with functions that include responsibility for, coordinating whole-of-government policy, program and service delivery across the Australian Government and between other levels of government and the private sector. [10]

Under these new arrangements, it is intended that all Australian government departments will contribute to a single coordinated budget submission for Indigenous-specific programs. [10] This process is untested, and it is at the leading edge of these reforms. Over the last decade significant growth in Aboriginal health program funding as been achieved within the context of the health portfolios budget process. [31] It remains to be seen as to whether this can be sustained with initiatives in Aboriginal health competing with those in other sectors such as housing, education etc.

Regionally, the "joined up government' agenda in Aboriginal affairs is supported through the creation of Indigenous Coordination Centers (ICCs). It is intended that the ICCs will provide Indigenous Australians with a single point of contact with Australian government departments. Accordingly, each ICC is described as a 'whole of Australian government office', with staff from multiple agencies, headed by a manager responsible for developing the relationship with Aboriginal communities and coordinating the efforts of other government agencies. The ICCs are responsible for the development of SRAs. [10,32]

The formal connection between the ICCs and the Commonwealth Department of Health and Ageing has evolved slowly. Currently, there are about 21 health staff who have been assigned a relationship with 33 ICCs. Health staff have been co-located within an ICC in only a few instances, and a number cover more than one ICC (Office for Aboriginal and Torres Strait Islander Health, personal communication.).

## Indigenous health strategy and planning since 1995

Since 1995 the key elements of the national planning framework that has been established for Indigenous health includes:

• The National Strategic Framework for Aboriginal and Torres Strait Islander Health which was signed off by the Australian Health Ministers Conference in 2003. This strategy outlines the agreed priorities and key result areas for national development in Indigenous health [20,21].

• The agreement to an Aboriginal Health Performance Measurement framework that is aligned with the National Strategic Framework. [33]

• Framework Agreements in Aboriginal and Torres Strait Islander Health, which are intergovernmental agreements that provide the basis for collaborative service planning and development.

• Joint Planning Forums, which have been established at a jurisdictional level under the Framework Agreements and are responsible for the development of State and regional Aboriginal and Torres Strait Islander health plans.

The regional planning process has been critical to aligning health planning with nominated priorities and identified gaps in services. They have played a significant role in brokering agreements between different levels of government and the ACCHS sector. The development of the infrastructure and resources for Aboriginal and Torres Strait Islander primary health care is a high priority in both Commonwealth health policy and in nationally agreed strategy. [20,21] In this context, the ACCHS and other primary health services play a significant role, both as a focus for capacity building, but also a vehicle for the delivery of disease- and risk-based strategies.

## The Mulan Agreement

The 'Mulan Agreement' was one of the first SRAs to be developed. Mulan is a small Aboriginal community, population 150, 400 kilometres southwest of Halls Creek on the edge of the Great Sandy Desert in the remote northwest of Australia. The proposed agreement, dubbed the 'Hygiene Pact', attracted controversy in the Australian media. [34-38] Some, such as Patrick Dodson, said it represented a "return to native welfare days". [38] Other press reports pointed out that the local community (through the Catholic school) had already initiated a hand and face-washing program 18 months earlier in order to improve the control of trachoma eye disease. It has been suggested that this had been used to gain leverage in this SRA to get the Australian Government to pay for the installation of a petrol bowser. [39] I will return to this significant fact below.

This particular SRA has two goals:

• *To strengthen the Mulan community economy through the installation of fuel bowsers that will provide income to the local store and improve tourism opportunities; and*

• *Improve health within the community, particular among children, through the implementation of strategies to reduce the incidence of trachoma, secondary skin infection and worm infestations*. [40]

Here I will focus on the health component, and specifically that which is related to trachoma control as this aspect of the deal attracted most of the debate.

In this SRA, the Mulan community was entitled to a discretionary benefit of $A172,260 to pay for the installation of petrol bowsers (the University of Melbourne's Agreements, Treaties and Negotiated Settlements Database records a slightly higher figure of $221,000). [35,41] The Western Australian State government for its part was to ensure that children were tested regularly for trachoma, skin infection and worms and to monitor health services.[40] In return for their discretionary benefit the text of the draft agreement stipulated that the Mulan community would:

• *through the Council, and in conjunction with the Community Consulting Agents and the Clinic, start and keep up a program to make sure kids shower every day, wash face twice a day;*

• *Through the CDEP program [Community Development Employment Program] ensure that rubbish bins are at every house and that they are emptied twice each week;*

• *Through the housing manager ensure that household pest control happens four times each year;*

• *Through the ESO [Essential Services Officer] ensure that the rubbish tip is properly managed;*

• *Work with the Community Consulting Agents to develop and put in place strategies to ensure that petrol sold through the store is no used for petrol-sniffing; and*

• *Work with the Community Consulting Agents to monitor and report on the extent to which the community, family and individual commitments set out in this agreement are met*.

Further the agreement specifies that families and individuals in the Mulan Community will:

• *Make sure kids get to school, crèche and the clinic when they should;*

• *Make sure kids shower daily and wash faces twice daily*

• *Keep homes and yards clean and rubbish free and ensure that household rubbish gets into bin straightway – not on ground or in houses*

• *Ensure all households rents are paid so that Council can afford pest control, repairs (eg plumbing) and cost of rubbish removal*. [40]

## Trachoma control programs

The face-washing component of the Mulan SRA was specific to the control of eye disease caused by trachoma. Trachoma is an infectious disease of the conjunctiva, or lining of the eye, which has both an acute phase (manifest in early childhood) and chronic outcomes – the most significant of which is adult blindness. In Australia, the disease is now almost exclusively an Aboriginal and Torres Strait Islander disease – having been absent for over a century in the broader population. This decline in the broader community occurred prior to the introduction of trachoma control programs and is largely attributed to the improvements in housing and environmental infrastructure. [42]

Current national guidelines for trachoma control are based on a strategy endorsed by the World Health Organization and they emphasise a of range activities summarised by the acronym SAFE or: S(urgery); A(ntibiotic control); F(acial cleanliness); E(nvironmental prevention). In sum, the SAFE acronym articulates a multi-faceted strategy with distinct components. [42-44]

Environmental health strategies for trachoma control include activities that aim to improve the quality of housing, and environmental health hardware such as water supply, sewerage systems, garbage disposal etc. This component of a trachoma strategy is argued to be fundamental to long-term control on the basis of compelling circumstantial evidence. [43] Direct evidence for environmental measures in trachoma control, however, is limited to a small study that documented the impact of a fly control program. [43,44] Face-washing programs have been developed because of an observed correlation between facial cleanliness and active trachoma. However, the evidence that links such programs and improved community trachoma prevalence is weak. [43] National guidelines also advocate a range of antibiotic regimes for the treatment of infected and un-infected individuals depending local epidemiology of the disease. [42] Finally, surgical treatment is recommended for chronic trachoma (trichiasis) as this has shown to be effective in the prevention of blindness. [42,44] All strategies depend on the local delivery of a range of health and public health services by an appropriately skilled workforce.

In summary, effective trachoma control requires an integrated primary health and public health approach to its management and control with a:

*combination of screening activity and antibiotic and surgical treatment, and environmental improvements to promote personal and community hygiene*. [44]

## The Mulan SRA and trachoma control

The Mulan SRA deals with only one component of the comprehensive strategy required to control trachoma. It focuses on the behaviour of individuals and the local community council. The evidence to support its particular focus on face-washing activities is limited and at best indirect. The agreement does not address the delivery of the health, public health services, housing and environmental programs, which are integral to comprehensive trachoma control programs. It is silent on critical issues including which government agencies are responsible for the planning, development and delivery of these services.

In one sense this is to be expected as SRAs are in general designed to focus on the provision of a discretionary benefit. Unlike standard service agreements, SRAs do not specify the relationship between funding inputs, service activity or outputs and outcomes. Nevertheless, the success of this Mulan SRA (in terms of trachoma control) depends on the appropriate funding and coordination of a range of broader activities and services. The link between the local ICC and the regional planning process established in health are significant in this regard. It was made clear as a result of questions raised at the Senate Estimates hearings that officers from the Commonwealth department of health who managed the Indigenous eye health program did not provide advice on the development of this agreement, although there was input from the regional public health Unit of the Western Australia Health Department. [45]

Trachoma control is complex and if SRAs are to tackle health problems such as this, the local ICC needs to be able to access public health expertise to ensure that they are based on current evidence and best practice. Taking into account the limited scope of this SRA, it seems broadly consistent with current guidelines, although it could be argued that the focus on the hygiene practices of Aboriginal children would be better broadened to family activities. However, this issue raises broader questions as to how ICC managers establish health priorities with local communities. Is a focus on trachoma control the appropriate priority given the range of health issues confronting remote communities? To that end, it is critical that mechanisms are established to ensure that ICC managers have access to the necessary public health expertise to guide decision-making.

The negotiation of an SRA such as this may well provide a focus for consolidating activity that addresses local health priorities. In a context in which local leadership has focused a community on its responsibilities, this may provide an opportunity to support local success. In this instance there is good evidence that there was significant community leadership in negotiating this deal. However, it should also be noted that there is no evidence to suggest, at this stage, that the provision of discretionary benefits will influence individual behaviours in a sustained way. In this respect the SRA is distinct from the application of mutual obligation to individual welfare transfer payments where the threatened removal of benefits can act as a powerful incentive. It seems doubtful that if a SRA was negotiated with a non-Aboriginal community that the provision of some new infrastructure, such as roundabout or swimming pool, would be a sufficient incentive to cause sustained individual behavioural change. It is not unreasonable to ask whether the installation of a petrol bowser will similarly form an incentive for sustained behaviour change.

## Monitoring and evaluation

Given the untested assumptions that underpin this new approach, it is important that the efficacies of these arrangements are tested through rigorous monitoring and evaluation processes.

The Mulan agreement was first announced in December 2004. Yet by April 2005 there were reports in the press that the prevalence of Trachoma in Mulan had decreased from a high of 70 per cent in 2004 to zero. [46,47] However, trachoma prevalence data is collected in this region at the same time every year (around September). This is done in order to minimise the impact of seasonal variation on prevalence data. Prevalence data fluctuates each year, depending on a range of factors including seasonal changes (it is more common in dusty dry conditions) and population mobility (in a small community the absence of one family may have a significant impact on rates) (see Table 1). The high prevalence of trachoma in 2003 (70 per cent) was probably the result of an unusually dry year, and this probably also accounted for the increase in prevalence (to 58 per cent) which occurred in 2005 following the announcement of the Mulan agreement. Nor could the SRA had any impact on the low prevalence (16 per cent) that was measured in 2004, given that the agreement was not negotiated until after the annual screening had taken place. Trachoma prevalence had never reached zero in Mulan as was claimed in *The Australian*, and it is the long-term trend data that is more significant here, not the dips and falls between years.

Press commentary on this agreement has fluctuated from premature claims of success to equally premature claims of failure. Politicians on all sides have exploited the various reports of prevalence data. When given the opportunity, the then Minister for Aboriginal Affairs, Senator Amanda Vanstone, did not correct the premature claims that were circulating in the press in April 2005. [47] Whether she was misinformed or mischievous is probably moot. Certainly, when it became apparent that the prevalence data for 2005 had in fact risen, Opposition Senators also took the opportunity to score political points during the Senates Estimates process.[48] The political stakes in this new agenda are clearly high. This only further underscores the need for rigorous independent evaluation.

## Commentary

There are a number of key issues that need to be addressed if SRAs are to be effective for Indigenous health strategy. In the first instance, it seems unlikely to work if they were to be used as vehicles for a radical agenda to erase Indigenous self-determination. In the second, critical issues between the relationship between these agreements and Indigenous health planning need to be addressed.

SRAs were established to signal a move away from the principles of self-determination in Aboriginal policy. The abolition of ATSIC was further emblematic of the desire of the Howard government to engage with Aboriginal Australia without the mediating influence of representative structures or bodies. However, it is difficult to imagine how a SRA could be effectively negotiated without the input of a local Aboriginal leadership, organisation or representative structure playing a role in the process of negotiation. To the extent that it has been successful, the Mulan SRA demonstrates the importance of an organised local leadership. It does not seem possible, without developing a new process, how local priorities would be understood or communicated.

It could be further argued that regional and national peak policy bodies continue to play a role in successful implementation of local agreements. The relationship between the National Aboriginal Community Controlled Health Organisation (the peak body representing ACCHs) and the health portfolio has deteriorated over recent years. Yet this continues to be a source of expert advice on clinical, population health and service issues in Aboriginal health. The current national guidelines for Trachoma control were developed with the input of NACCHO, which has the potential to mobilise the expert knowledge and experience of workers in this sector. It would be counter to the success of government strategy to completely erase such institutional manifestations of self-determination.

More significantly, the development of a partnership with the Aboriginal community controlled sector has been foundational to most national strategy in Aboriginal health. [20,21] This sector plays a significant role in the delivery of primary health care services to Aboriginal and Torres Strait Islander people – it is likely, for pragmatic reasons alone, that effort will still need to be placed on the development of capacity in this component of the health system. Notwithstanding the significant role of mainstream primary health services, Indigenous-managed services continue to play a key role in this arena.

If locally agreed SRAs, which focus on health outcomes, are to be successful they need to articulate with established processes in Indigenous health strategy. Health gain in nearly all instances requires more than simple individual behavioural change. If the Mulan agreement is to have an effect in terms of health gain, behavioural change needs to be supported by the provision of effective primary health and environmental health services. Health planning, service development and workforce development need to align with these local priorities. It is acknowledged that some steps have been taken to connect the ICCs with the health portfolio, but the question remains as to whether this is adequate.

Whilst a focus on the development of relationships between ICCs and regional planning processes will be critical, it is also important that the development of SRAs also reflects best practice and evidence about effective disease control. It would be inefficient to establish regional processes to collate, synthesise and analyse evidence of this kind – so the development of broader national mechanisms is needed to inform the decision-making of the managers of ICCs. Where best practice guidelines have been developed, these need to be made available to ICC managers. ICC managers need some support in identifying other sources of public health advice. They also need to be across established national priorities for Indigenous health.

The new arrangements do provide opportunities to progress the development of an inter-sectoral agenda in health. Notwithstanding the ideological commitment of some for a more radical agenda, local Indigenous leadership, Indigenous-managed-services, regional bodies and policy organisations will continue to play a role in the roll-out of strategy. Further, if these new arrangements are to be effective articulations need to be created in to link these processes more effectively with health planning and expert advisory processes. Whilst these issues are clearly critical to good policy development, it is also important that these agreements do not reinforce a sense of failure in local communities. In that sense there is probably much more at stake for communities such as Mulan than the political fortunes of the advocates and critiques of these new arrangements. The articulation of SRAs with health strategy and planning is critical ands deserves a close focus in the planned review of SRA's.

## Abbreviations

ACCHS Aboriginal Community Controlled Health Services

ATSIC Aboriginal and Torres Strait Islander Commission

ATSIS Aboriginal and Torres Strait Islander Services

CDEP Community Employment Development Program

ESO Essential Services Officer

SRA Shared Responsibility Agreement

ICC Indigenous Coordination Centre

NACCHO National Aboriginal Community Controlled Health Organisation

OIPC Office of Indigenous Policy Coordination

## Competing interests

The author(s) declare that they have no competing interests.

**Table 2:** Trachoma Prevalence: Mulan. Source: [49]

**Year**	**1997**	**1998**	**1999**	**2000**	**2001**	**2002**	**2003**	**2004**	**2005**
**Prevalence %**	17.9	40.9	30.7	24.3	32	27.4	75	16	56
